# First identification of occult hepatitis B infection among ethnic minority students in Thai Nguyen, Vietnam

**DOI:** 10.1186/s12879-025-12347-7

**Published:** 2025-12-23

**Authors:** Vu Nhi Ha, Le Chi Cao, Tran Hai Dang, Duong Thi Phuong Thao, Dao Thi Huyen, Tran Thi Thu Hien, Tran Thi Thanh Huyen, Nguyen Xuan Vu, Nguyen Tien Dung, Duong Van Cuong, Le Huu Song, Nguyen Linh Toan, Truong Nhat My, Thirumalaisamy P. Velavan

**Affiliations:** 1 Thai Nguyen University of Medicine and Pharmacy, Thai Nguyen, Vietnam; 2 Institute of Tropical Medicine, University of Tübingen, Wilhelmstrasse 27, 72074 Tübingen, Germany; 3 Hue University of Medicine and Pharmacy, Hue University, Hue, Vietnam; 4 Thai Nguyen University of Agriculture and Forestry, Thai Nguyen, Vietnam; 5 Vietnamese German Center for Medical Research, VG-CARE, Hanoi, 10000 Vietnam; 6 108 Military Central Hospital, Hanoi, 10000 Vietnam; 7 Vietnam Military Medical University, Hanoi, Vietnam; 8 Faculty of Medicine, Duy Tan University, Danang, Vietnam

**Keywords:** Occult hepatitis B, HBV genotype B, Ethnic minority, HBV serological markers

## Abstract

**Background:**

Hepatitis B virus (HBV) remains a major health concern particularly in regions with intermediate-to-high endemicity such as Vietnam. Occult hepatitis B infection (OBI) defined as the presence of replication-competent HBV DNA in the absence of detectable hepatitis B surface antigen (HBsAg) poses challenges for diagnosis and blood safety. However, data on OBI among Vietnam’s ethnic minority populations are scarce.

**Methods:**

A cross-sectional study was conducted among 267 HBsAg-negative ethnic minority students at Thai Nguyen University of Medicine and Pharmacy in 2024. Serum samples were screened for HBV serological markers (anti-HBs, anti-HBc) using commercial ELISAs. HBV DNA was extracted, amplified by nested PCR targeting a conserved S/P region, and sequenced for genotyping and mutational analysis. Quantitative real-time PCR (qPCR) was used to assess viral loads.

**Results:**

Among participants, 57% were susceptible (anti-HBs–/anti-HBc–), 27% exhibited vaccine-induced immunity, 12% had resolved infections, and 4% showed isolated anti-HBc positivity. Overall, 16% were anti-HBc positive, indicating prior exposure. HBV DNA was detected in two samples (0.7%), both with undetectable viral loads by qPCR. One case represented seronegative OBI and the other seropositive OBI. Phylogenetic analysis classified both isolates as genotype B. Mutational analysis identified substitutions K122R, F200Y, Y206C, and I187V, with S117N uniquely present in one isolate.

**Conclusions:**

This study provides the first evidence of OBI among ethnic minority students in northern Vietnam. Although prevalence was low, the high proportion of HBV-susceptible individuals highlights ongoing vulnerability and underscores the need for strengthened immunization, awareness, and surveillance programs in underserved communities.

**Supplementary Information:**

The online version contains supplementary material available at 10.1186/s12879-025-12347-7.

## Introduction

Hepatitis B virus (HBV) remains a major global health concern, with an estimated 254 million people living with chronic infection in 2022, leading to 1.1 million deaths and 1.23 million new infections annually [1]. Despite this substantial burden, only 13.4% of chronic HBV cases are diagnosed, and merely 2.6% receive antiviral therapy [1]. Vietnam is among the nine countries contributing most to the global HBV burden, with an estimated hepatitis B surface antigen (HBsAg) prevalence of 6.5% in 2022 [1]. A nationwide meta-analysis reported a decline in HBsAg prevalence among blood donors between 2011 and 2020 (to 1.4%); however the rates remain high among high-risk groups, reaching 9.4% in hemodialysis patients and 8.6% in surgical patients [2].

Beyond overt infection, HBV can persist in replication-competent form among individuals who test negative for HBsAg, a condition referred to as occult hepatitis B infection (OBI). OBI is defined by the presence of HBV DNA in liver tissue and/or blood despite undetectable HBsAg using conventional tests [3]. Because low-level viraemia and S-gene mutations can evade standard diagnostic tests, OBI remains underrecognized. This diagnostic gap contributes to residual risks of HBV transmission via blood transfusion and viral reactivation during immunosuppressive therapy. Routine OBI screening can therefore enhance transfusion safety, guide prophylaxis, and support HBV elimination goals [3].

Although chronic HBV infection has been extensively studied in Vietnam, data on OBI are scarce. A prior investigation among blood donors in Hanoi reported an OBI prevalence of 0.3% [4], but information on ethnic minority populations is lacking. These communities, often residing in remote areas, face limited healthcare access, lower vaccination coverage, and reduced awareness of HBV prevention. Understanding OBI in such populations is crucial for designing equitable elimination strategies. Therefore, this study aimed to determine the prevalence of OBI and characterize the HBV serological profile among ethnic minority adults in Thai Nguyen province in Vietnam.

## Materials and methods

### Study cohort

A cross-sectional study was conducted at the Thai Nguyen University of Medicine and Pharmacy (TUMP) in 2024. Serum samples (*n* = 267) were collected from healthy HBsAg-negative students belonging to ethnic minority groups of Tay, Nung, Thai, Mong, Muong, Dao, San Diu and others over a three-day recruitment period. Sample size estimation was performed using the standard formula for cross-sectional prevalence studies at 95% confidence, applying an expected OBI prevalence of 9% derived from regional Southeast Asian data [5]. This yielded a required minimum sample size of approximately 126 participants. During the recruitment period, 267 eligible ethnic minority students were enrolled, exceeding the required number and ensuring adequate statistical precision for estimating OBI prevalence in this population. Demographic information, including age, sex, ethnicity, and place of residence, was collected using standardized questionnaires developed specifically for this study (Supplementary Data).

### Screening of HBV serological markers

All serum samples were screened for HBV markers using commercial ELISA assays, including anti-HBs (Monolisa™ anti-HBs PLUS, BIO-RAD, Hercules, CA, USA) and anti-HBc (Monolisa™ total anti-HBc PLUS, BIO-RAD, Hercules, CA, USA). Anti-HBs titters were quantified, with seropositivity defined as > 10 mIU/mL. Anti-HBc was assessed qualitatively, and results exceeding the manufacturer’s cut-off value were classified as positive. The analytical sensitivity and specificity of all assays were reported to be greater than 99%. Serological profiles were classified as: (a) susceptible (anti-HBs–/anti-HBc–), (b) vaccinated (anti-HBs+/anti-HBc–), (c) past infection/resolved (anti-HBs+/anti-HBc+), and (d) potential OBI (anti-HBs–/anti-HBc+).

### Nucleic acid isolation

Viral DNA was extracted from 200uL of serum using the QIAamp DNA Mini Kit (Qiagen GmbH, Hilden, Germany) following the manufacturer’s protocol. DNA concentration and purity were measured using a NanoDrop™ spectrophotometer (Thermo Fisher Scientific, Waltham, MA, USA). Extracted DNA was stored at -80 °C until molecular analysis.

### Qualitative nested PCR, sanger sequencing and quantitative real-time PCR

A nested PCR was performed to amplify a conserved 332-bp fragment of the HBV surface/polymerase (S/P) region, following previously published protocols [6]. The amplification was performed in two rounds using primer sets targeting conserved regions of the HBV genome. Outer PCR was carried out in 25 µL reaction volume (1x PCR buffer, 0.2 mM dNTPs, 1.5mM MgCl2, 0.2 µM each primer, and 0.5U Taq DNA Polymerase (Qiagen GmbH, Hilden, Germany), with primers HBV-022, HBV-065, and HBV-066; 35 cycles consisting of denaturation at 94 °C for 30 s, annealing at 55 °C for 30 s, and extension at 72 °C for 30 s. Inner PCR was carried out in 25 µL reaction volume (1x PCR buffer, 0.2 mM dNTPs, 1.5mM MgCl2, 0.2 µM each primer, and 0.5U Taq DNA Polymerase (Qiagen GmbH, Hilden, Germany), with primers HBV-024, HBV-041, and HBV-064; 30 cycles consisting of denaturation at 94 °C for 30 s, annealing at 63 °C for 30 s, and extension at 72 °C for 30 s. Each run included a positive and a negative control to ensure assay validity. The amplicons were run on agarose gel electrophoresis, on 1.5% agarose gels stained with SYBR green and visualized under UV illumination. Bands of the expected size were purified using ExoSAP-IT PCR (Thermo Fisher Scientific, Waltham, MA, USA). Purified amplicons were sequenced bidirectionally using the BigDyeTM Terminator v.3.1 Cycle Sequencing Kit (Thermo Fisher Scientific, Waltham, MA, USA) on an Applied Biosystems 3130xl Genetic Analyzer (Applied Biosystems, Beverly, MA, USA).

Quantitative real-time PCR (qPCR) was performed to assess HBV DNA levels using the SensiFAST™ one-step RT-PCR kit (Meridian Biosciences, USA) on a Light Cycler 480 system (Roche, Switzerland), under the standard cycling conditions described previously [6]. Results were expressed in international units per milliliter (IU/mL). The assay detection limit was 10 IU/mL.

### Sequence analysis

Raw sequences were trimmed in SeqMan v6.1 (DNASTAR, USA) and aligned with 37 reference sequences (HBV genotypes A–J) retrieved from the NCBI GenBank database using BioEdit v7.2.6. Phylogenetic analysis was conducted in MEGA 11 using the neighbor-joining algorithm with the kimura-2 parameter model plus gamma and invariant sites (K2 + G + I), supported by 1000 bootstrap replicates. The tree was visualized with iTOL (https://itol.embl.de/). Mutational analysis of the surface (S) gene and reverse transcriptase (RT) domain was conducted using BioEdit v7.2.6 and Geno2pheno (https://hbv.geno2pheno.org) online platform. Sequences were submitted to GenBank (Assigned accession numbers: PX406156-PX406157).

### Statistical analysis

All statistical analyses were performed using SPSS 20.0 (IBM Corp., Armonk, NY, USA). Continuous variables were summarized as medians with interquartile ranges (IQRs), and categorical data as frequencies and percentages. Comparison between groups used the Chi-square or Fisher’s exact tests for categorical variables and the t-test or Kruskal–Wallis test for continuous data. A two-sided p-value < 0.05 was considered statistically significant.

## Results

### Baseline characteristics

A total of 267 HBsAg negative serum samples were obtained from healthy students aged 20–45 years (median = 22 years; IQR = 21–24). Female participants comprised 70% (*n* = 186), and males 30% (*n* = 81). Most students originated from rural areas (85%, *n* = 226). The predominant ethnic groups were Tay (41%, *n* = 109), Nung (18%, *n* = 48), and Thai (11%, *n* = 30), with other minorities collectively representing < 10%.

### HBV serological profile

ELISA screening revealed heterogeneous HBV serological patterns among the 267 participants (Table 1). The majority were susceptible to infection, with 57% (151/267) negative for both anti-HBs and anti-HBc. Vaccine-induced immunity (anti-HBs⁺/anti-HBc⁻) was observed in 27% (73/267), while resolved past infection (anti-HBs⁺/anti-HBc⁺) accounted for 12% (33/267). A small subset (4%, 10/267) exhibited isolated anti-HBc positivity, representing potential cases of occult HBV infection (OBI). Overall, 16% (46/267) of participants were anti-HBc positive, indicating prior HBV exposure. Comparative analyses by sex, ethnicity, and region of residence revealed no statistically significant differences in serological patterns (*p* > 0.05) (Table 2).

**Table 1:** Anti-HBs and anti-HBc seroprevalence among the tested individuals from various ethinic minorities

Serology	Percentage (*n*)
Susceptible (anti-HBs–/anti-HBc–)	151/267 (57%)
Vaccinated (anti-HBs+/anti-HBc–)	73/267 (27%)
Resolved infection (anti-HBs+/anti-HBc+)	33/267 (12%)
Potential OBI (anti-HBs–/anti-HBc+)	10/267 (4%)

**Table 2:** Comparison of anti-HBs titers and anti-HBc positivity across different ethnic groups and regions of residence

Classification	Anti-HBc positivity*	Anti-HBs positivity*	Anti-HBs titer (mIU/ml) Mean (± SD)^#^
**Ethnicity**			
Tay (*n* = 109)	12%	39%	68 ± 135
Nung (*n* = 48)	15%	52%	62 ± 130
Thai (*n* = 30)	27%	37%	83 ± 144
Mong (*n* = 18)	33%	44%	97 ± 156
Muong (*n* = 15)	27%	40%	88 ± 150
Dao (*n* = 14)	14%	36%	39 ± 105
San Diu (*n* = 11)	0%	18%	8 ± 13
Others (*n* = 22)	14%	27%	51 ± 119
*p-value*	*p* > 0.05	*p* > 0.05	*p* > 0.05
**Region of Residence**			
Rural areas (*n* = 226)	16%	40%	69 ± 133
Urban areas (*n* = 41)	15%	37%	54 ± 130
*p-value*	*p* > 0.05	*p* > 0.05	*p* > 0.05

* Chi-square test was used to compare seropositivity between groups

^#^ Kruskal–Wallis test was used to compare mean of anti-HBs titer

### HBV DNA detection and genotyping

**Fig. 1:**
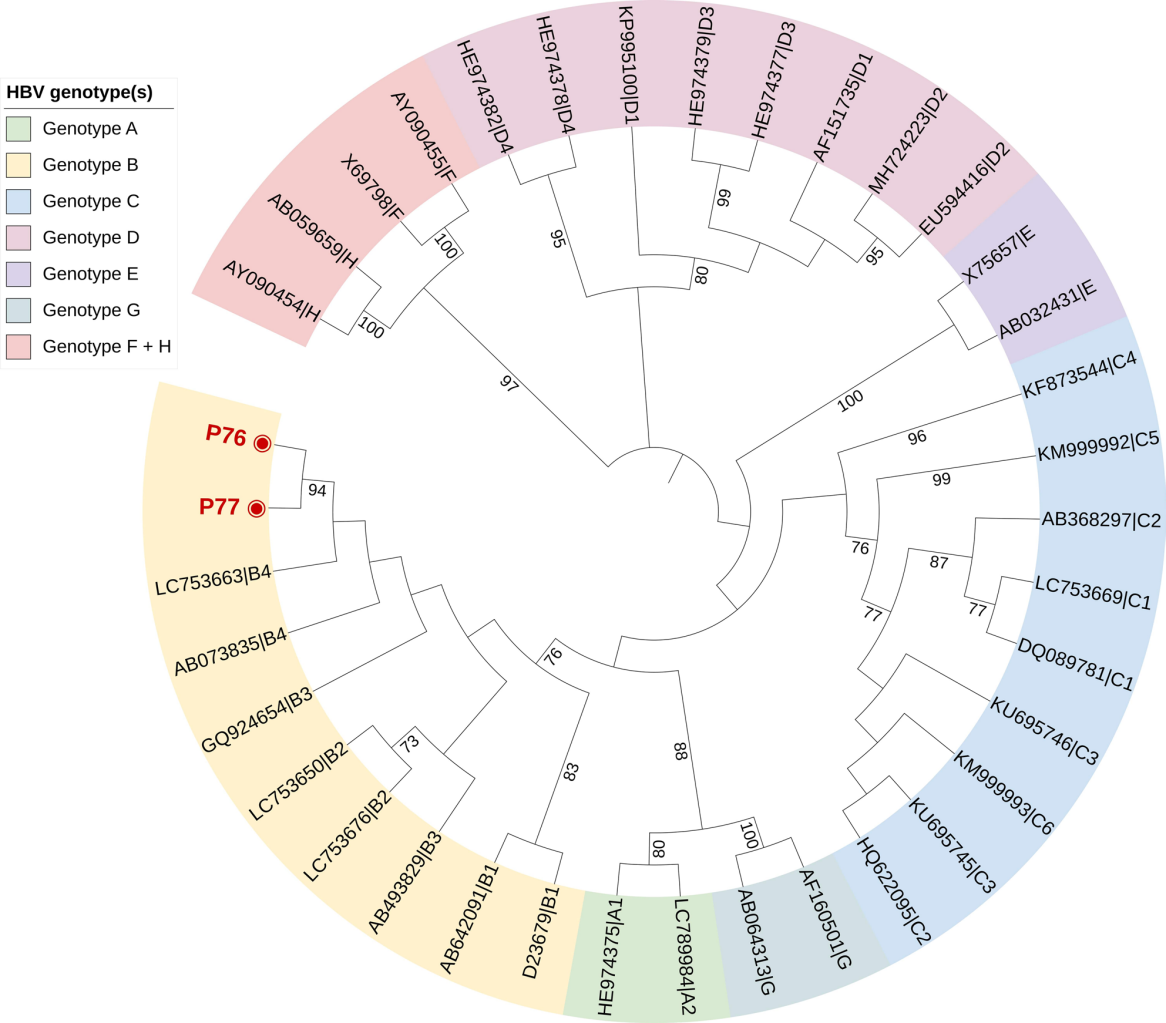
Phylogenetic tree of HBV based on the partial S/P region. The tree was constructed using the K2 + G + I model with 1,000 bootstrap replicates. Reference sequences representing genotypes and subgenotypes A–H were retrieved from the NCBI HBV database. The study sequence is shown in bold with a filled circle

Nested PCR identified HBV DNA in two samples (P76 and P77), corresponding to an OBI prevalence of 0.7% (2/267). Both samples showed undetectable viral loads by quantitative PCR (< 10 IU/mL). Sample P76 was negative for both anti-HBs and anti-HBc, consistent with a seronegative OBI, whereas sample P77 was positive for anti-HBs (342 mIU/mL) but negative for anti-HBc, indicating a seropositive OBI profile. Phylogenetic analysis clustered both isolates within genotype B, the predominant genotype in Vietnam (Fig. 1). Mutational analysis revealed identical amino acid substitutions: K122R, F200Y, and Y206C, in the surface region and I187V in the reverse transcriptase domain. An additional mutation, S117N, was detected in P76 within the major hydrophilic region of HBsAg, a site frequently associated with immune escape (Table 3).

**Table 3:** HBV reverse transcriptase (RT) and surface (S) mutations with serological markers in two OBI cases

ID	anti-HBs	anti-HBc	Mutations in the (S) gene	Mutations in the (RT) domain
P76 (GenBank ID: PX406156)	Negative	Negative	S117N, K122R, F200Y, Y206C	I187V
P77 (GenBank ID: PX406157)	Positive (342 mIU/ml)	Negative	K122R, F200Y, Y206C	I187V

## Discussion

In Vietnam, where HBV remains of intermediate to high endemicity, identifying OBI is essential for breaking residual transmission chains and improving blood-safety programs [4, 7]. The prevalence of OBI varies markedly depending on assay sensitivity, molecular methods, and study populations. Reported rates range from 0.013% in China [8] to 11% in healthy HBsAg-negative individuals in Laos [9]. This study finding at 0.7% OBI prevalence among ethnic minority students in Thai Nguyen aligns with the 0.3% rate previously reported among Vietnamese blood donors [4], and is considerably lower than the overall Asian average of 4% [5]. This suggests that, within this young and apparently healthy cohort, the risk of HBV transmission from HBsAg-negative individuals in ethnic minority population is limited. Nevertheless, targeted OBI screening, and sustained vaccination outreach could further strengthen surveillance and prevention efforts in underserved areas.

Serological profiling revealed heterogeneous exposure and immunity patterns. Overall, 16% of participants were anti-HBc positive, similar to rates reported among students in DaNang from central Vietnam (17.6%) [10] but lower than among blood donors in Hanoi, in northern Vietnam [4]. A representative 27% were anti-HBs positive but anti-HBc negative, indicating vaccine-induced immunity, whereas more than half (57%) lacked both markers, representing a large susceptible pool. The predominance of seronegative individuals underscores persistent immunization gaps among ethnic minorities and young adult populations often missed by early childhood vaccination campaigns. Integrating booster doses and HBV education initiatives into university health programs could help bridge these immunity gaps [11].

Two OBI cases were identified. Sample P76 was seronegative for both anti-HBs and anti-HBc, a rare pattern seen in 10–20% of OBI cases worldwide [3], while sample P77 exhibited anti-HBs positivity without anti-HBc. Both belonged to genotype B, consistent with the predominant genotype in Vietnam [12]. Genotype B infections typically exhibit lower viral loads and earlier HBeAg seroconversion than genotype C [13], which may partly explain the undetectable viral loads observed by qPCR. Both cases were female, aged 33 and 35 years, respectively, and originated from the Tay ethnic community in rural areas. However, no conclusion can be drawn regarding an elevated OBI prevalence in this community, as Tay is the predominant ethnicity in the study population, and the overall anti-HBc seropositivity in this group is comparable to that of other ethnic groups. Several amino acid substitutions were observed in both isolates, including F200Y and Y206C in the surface region, S117N in sample P76, and I187V in the reverse transcriptase domain. The Y206C substitution has been associated with lower HBsAg titers [14], while S117N may alter antigenicity within the major hydrophilic region [15]. Although K122R was also detected, this substitution represents a serotype-specific polymorphism within the “a” determinant of genotype B strains prevalent in Vietnam and has not been shown to significantly affect HBsAg secretion or contribute directly to OBI [16]. Furthermore, the I187V substitution identified in both OBI cases may impair viral replication without affecting in vitro drug sensitivity [17].

While our detection of OBI in healthy population with low immediate risk, it is important to note that even low-level or “occult” HBV infection may carry long-term risks. Chronic HBV infection including forms with low or intermittent viremia can lead over time to progressive liver fibrosis, cirrhosis, and hepatocellular carcinoma, especially when compounded by other risk factors such as pregnancy or co infection with other viral hepatitis [18–20]. Recent large cohort studies have documented high rates of cirrhosis and HCC among chronic HBV-infected individuals, underscoring that “silent” infections can eventually lead to severe liver disease even in the absence of overt symptoms [21]. This risk supports the notion that surveillance and early antiviral therapy remain medically relevant, especially in populations with limited access to regular follow-up or where comorbidities (e.g., metabolic conditions, fatty liver) may accelerate progression [22].

This study has limitations. The modest sample size and cross-sectional design, with only two OBI cases detected, restrict generalizability. Single time-point sampling may underestimate transient viremia, and lack of vaccination records limits interpretation of serological patterns. Future research should include multi-site cohorts from other ethnicities, longitudinal monitoring, and comprehensive vaccination histories to clarify OBI epidemiology and its clinical significance among Vietnam’s minority populations.

In conclusion, this study provides the first evidence of OBI among ethnic minority students in northern Vietnam. Although the prevalence is low, the high proportion of HBV-naïve individuals highlights ongoing vulnerability and underscores the need for strengthened immunization and surveillance strategies tailored to marginalized communities.

## Supplementary Information

Below is the link to the electronic supplementary material.

Supplementary Material 1

## Data Availability

All data generated or analyzed in this study are included in this article. The two OBI sequences obtained have been deposited in the NCBI GenBank database under accession numbers PX406156 and PX406157.
