# Supplementary material for: First identification of occult hepatitis B infection among ethnic minority students in Thai Nguyen, Vietnam
Source: BMC Infect Dis. 2025 Dec 23;26:146. doi: 10.1186/s12879-025-12347-7 (PMC12836949; doi:10.1186/s12879-025-12347-7)
Supplement: Supplementary file 1 — Supplementary Material 1 [file 12879_2025_12347_MOESM1_ESM.docx]

| ID:…………………….. | Thái Nguyên, *date month* *year* 2024 |
| --- | --- |

**SELF-COMPLETED QUESTIONNAIRE**

**The Burden of Emerging and Re-emerging infectious diseases in minority ethnic populations in Thai Nguyen Province, Viet Nam**

| **SECTION A. GENERAL PERSONAL INFROMATION** | | | |
| --- | --- | --- | --- |
| Q1 | Full name |  | |
| Q2 | Year of Birth |  | |
| Q3 | Ethnicity |  | |
| Q4 | Class |  | |
| Q5 | Gender | Male |  |
|  |  | Female |  |
| Q6 | Birthplace | Rural area |  |
|  |  | Urban area |  |
| Q7 | Educational level | High school |  |
|  |  | University |  |
|  |  | Postgraduate |  |
| **SECTION B. EXPOSURE INFORMATION** | | | |
| Q8 | Permanent residence | Rural area |  |
|  |  | Urban area |  |
| Q9 | Characteristic of residence | Mountainous area |  |
|  |  | Plain area |  |
| Q10 | Drinking water source | Well water |  |
|  |  | Tap water |  |
| Q11 | Livestock, Poultry, Wildlife Raising | Yes |  |
|  |  | No |  |
| Q12 | Contact with Household Pets | Yes |  |
|  |  | No |  |
| Q13 | Contact with Pigs, Wild Boars or Wildlife | Yes |  |
|  |  | No |  |
| Q14 | Contact with Other Animals (horses, sheep, cattle, goats, dogs, cats, etc.) | Yes |  |
|  |  | No |  |
| Q15 | Hunting wild animals | Yes |  |
|  |  | No |  |
| Q16 | Gardening | Yes |  |
|  |  | No |  |
| Q17 | Crop Cultivation | Yes |  |
|  |  | No |  |
| Q18 | Eating Raw or Undercooked Pig Liver | Yes |  |
|  |  | No |  |
| Q19 | Eating Sausages Made from Raw Liver | Yes |  |
|  |  | No |  |
| Q20 | Eating Raw Pig Sausages | Yes |  |
|  |  | No |  |
| Q21 | Eating Self-Made Sausages or Bacon Not Fully Cooked | Yes |  |
|  |  | No |  |
| Q22 | Eating undercooked wild meats (wild boar, deer) | Yes |  |
|  |  | No |  |
| Q23 | Eat raw seafood (sushi, sashimi, fish salad, shrimp salad,...) | Yes |  |
|  |  | No |  |
| Q24 | Eating Homegrown Vegetables | Yes |  |
|  |  | No |  |
| Q25 | History of blood transfusion | Yes |  |
|  |  | No |  |
| Q26 | History of Jaundice | Yes |  |
|  |  | No |  |
| Q27 | History of hepatitis B virus infection | Yes |  |
|  |  | No |  |
| Q28 | Type of Toilet Used | Specify |  |
| Q29 | When Do You Wash Hands with Soap | Before eating |  |
|  |  | Before cooking |  |
|  |  | After using toilet |  |
|  |  | Other (specify) |  |
| Q30 | There is a common garbage dump of other families around the house | Yes |  |
|  |  | No |  |
| Q31 | If yes, how often is waste collected | Specify |  |
| Q32 | During the last 6 Months, Has Your Family Raised Livestock or Poultry | Yes |  |
|  |  | No |  |
| Q33 | Do you use industrial feed in livestock farming? | Yes |  |
|  |  | No |  |
| Q34 | Please Name the Industrial Feed Used | Specify |  |
| Q35 | Do You Use Antibiotics in Animal Husbandry | Yes |  |
|  |  | No |  |
